# Fuzzy Regulator Design for Wind Turbine Yaw Control

**DOI:** 10.1155/2014/516394

**Published:** 2014-02-11

**Authors:** Stefanos Theodoropoulos, Dionisis Kandris, Maria Samarakou, Grigorios Koulouras

**Affiliations:** ^1^School of Engineering and Physical Sciences, Heriot Watt University, Edinburgh EH14 1AS, UK; ^2^Department of Electronic Engineering, School of Technological Applications, Technological Institute of Athens, 12210 Athens, Greece; ^3^Department of Energy Technology Engineering, School of Technological Applications, Technological Institute of Athens, 12210 Athens, Greece

## Abstract

This paper proposes the development of an advanced fuzzy logic controller which aims to perform intelligent automatic control of the yaw movement of wind turbines. The specific fuzzy controller takes into account both the wind velocity and the acceptable yaw error correlation in order to achieve maximum performance efficacy. In this way, the proposed yaw control system is remarkably adaptive to the existing conditions. In this way, the wind turbine is enabled to retain its power output close to its nominal value and at the same time preserve its yaw system from pointless movement. Thorough simulation tests evaluate the proposed system effectiveness.

## 1. Introduction

The joint consideration of both environmental concerns and economic issues has triggered an ever-increasing thoughtfulness on renewable energy resources. For instance, European Union aims to succeed by 2020 in decreasing greenhouse gas emissions by 20% (compared to 1990 ones), by increasing the amount of renewable energy to 20% and reducing the overall energy consumption by 20% through energy efficiency through the implementation of the 20/20/20 initiative [1].

A great part of the scientific research on renewable energy resources focuses on wind power, that is, the conversion of the energy coming from flowing air into electricity which is performed through the utilization of air turbines [2–5]. Wind power has numerous advantages when compared against fossil fuels, because it is an abundant, broadly distributed, and environmentally clean source of energy. Due to these advantages, the global installed capacity of wind power increases rapidly. For instance, it is expected that 20% of the USA electricity demand in 2030 will be provided by wind energy [6].

However, despite the mostly promising prospects of wind energy, there are still many issues regarding the efficient harvesting of wind power which should be effectively handled. More specifically, both the velocity and the direction of wind are highly variable parameters thus making practically infeasible the total exploitation of the current wind momentum. Additionally, although low wind results just in low power output, high velocity wind may cause severe failure of many of the mechanical components of the wind turbine [7].

These changes in the wind parameters may occur rapidly and in short time intervals. Consequently, the control of the wind turbine movement and reactions is needed to optimize its operation. Pitch and yaw control are the most common and effective ways that modern wind turbines utilize to increase power output [8].

The rest of this work is organized as follows. In Section 2, an analysis of the implementation of yaw control takes place. In Section 3, the system model adopted is described. The performance evaluation of the proposed fuzzy control system takes place in Section 4 through the description of the simulation tests and the corresponding results. Finally, Section 5 concludes the paper.

## 2. Yaw Control

In a wind turbine, which has the ability to orientate its rotor towards the wind, the so-called yaw system is the component responsible for this operation.

The simplest type of yaw control may be achieved through the use of the so-called passive yaw control systems which use the wind force so as to not only rotate the rotor but also orientate the wind turbine nacelle. The typical form of such systems consists of a roller bearing connecting the tower and the nacelle, along with a tail fin mounted on the nacelle which aims to apply an apt torque to the nacelle so as to turn the turbine rotor to the direction of wind. Passive yaw control offers a low cost solution which is effective mainly in small wind turbines.

On the other hand, more advanced control capabilities may be provided through the use of the so-called active yaw systems which are applied to the majority of modern medium and large sized wind turbines. The main feature which differentiates them from passive yaw systems is that they include mechanisms able to produce torques in order to suitably revolve the nacelle of the wind turbine according to the current wind parameters.

A typical active yaw system consists of a yaw bearing that provides the rotating joining between nacelle and tower, a yaw drive (yaw motor and gearbox), and the controller. In most cases, a yaw brake is also needed to provide a counter torque. Normally, wind direction signal originated from the wind sensor is the input to the controller. The yaw control system aligns the turbine's nacelle with the wind direction in order to minimize the yaw angle error, that is, the result of the subtraction between turbine main axis angle and wind direction.

While the yaw angle misalignment correction lies in the center of the yaw control schemes [7–12], certain issues must be considered so as to ensure the safety of the mechanical parts. For instance, the yaw system must not be extremely sensitive to movement, to avoid unnecessary wear on the mechanical components [7–9].

The control design depends on the choice of the motor (system actuator) due to the different operational characteristics of the types of motors. For instance, stepper motors are considered highly accurate actuators and in comparison to other DC or AC motors they do not need velocity control during their operation [13–15].

Three-term (PID) or other conventional feedback controllers are applied in numerous control applications. In the cases, where there is a mathematical model in hand, such controllers are considered to be particularly accurate. However, their nature, which is highly linear and symmetrical, obstructs their successful utilization in nonlinear circumstances. Heating and the production of wind power are typical examples of this type of applications. When dealing with such applications, other types of control systems like fuzzy logic, fuzzy-PID controllers, neural networks, or a combination of them are more suitable because they can handle nonlinear models more effectively [16–22].

Modern research works focus on the use of fuzzy logic in yaw control [23–27]. Some of them [23, 24] deal with the yaw control of vehicles. Others use fuzzy logic in order to select the values of the terms used in the PID control of wind turbine yaw angle [25, 26].

The research work presented in this paper focuses on the yaw control of wind turbines through the development of a fuzzy controller which is designated to perform adaptive regulation of the orientation of the wind turbine rotor towards the wind in correspondence with the wind parameters that exist in the area surrounding the wind turbine. The enhancement provided by the proposed control system is that, contrary to other systems which perform fuzzy yaw control [27], the controller developed takes into account both the wind velocity and the acceptable yaw error correlation in order to achieve maximum performance efficacy.

## 3. Simulation Model Description

**Figure 1:**
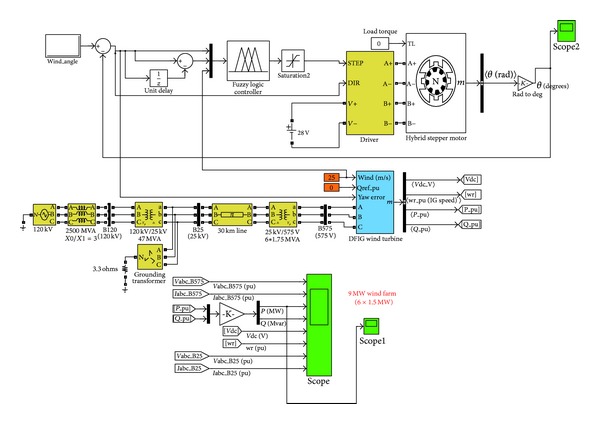
Overview of the simulation model adopted.

The simulation model adopted for the proposed yaw control system was built by using Matlab/Simulink environment. As shown in Figure 1, it consists of a two-phase permanent magnet stepper motor with its driver, a wind farm unit, and a fuzzy controller.

### 3.1. Stepper Motor

**Figure 2:**
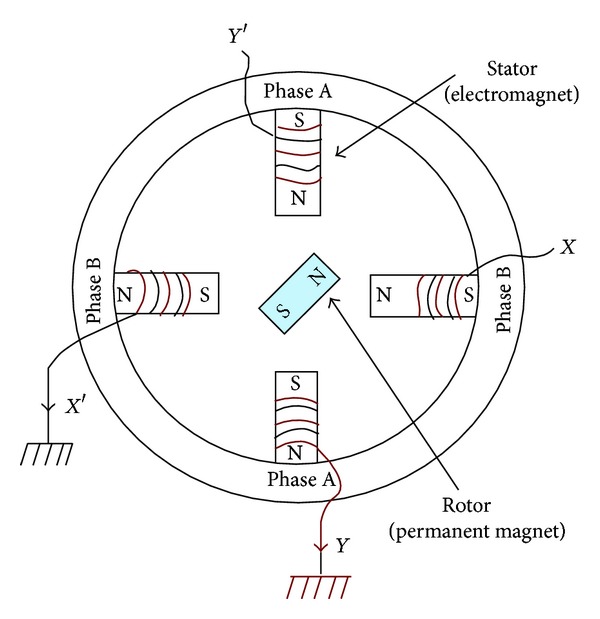
A two-phase PM stepper motor circuit schematic diagram.

The stepper motor which is incorporated in the aforementioned model is supposed to have its basic construction similar to that illustrated in Figure 2. It is also assumed that this stepper motor operates according to the model equations which are analytically given in [13–15].

Generally, a typical permanent magnet stepper motor has the positioning accuracy advantage and a higher torque/volume ratio when compared to variable reluctance motors.

Precisely, the rotor unit of the specific motor incorporated in the developed model utilizes the standard full step operation and rotates by 1.8° degrees per step.

The motor driver utilizes signal modifying blocks. These blocks are utilized aiming at converting constant signals into properly synchronized pulses for the motor to operate with. In this way, considerable accuracy in positioning is achieved.

**Figure 3:**
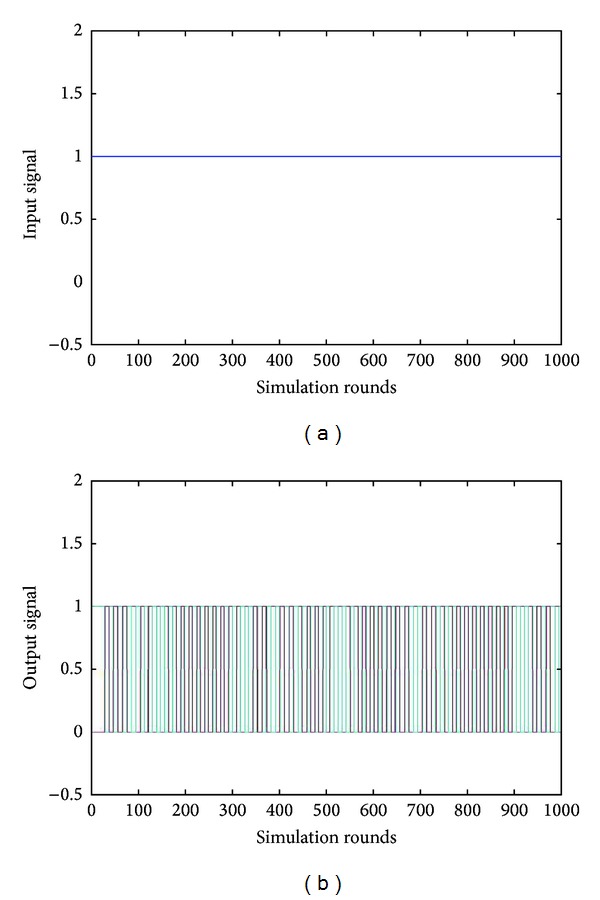
Constant signal transformation in pulses by the stepper motor driver.

Figure 3 depicts the pattern according to which this specific driver is designated to transform constant signals into a series of pulses.

**Figure 4:**
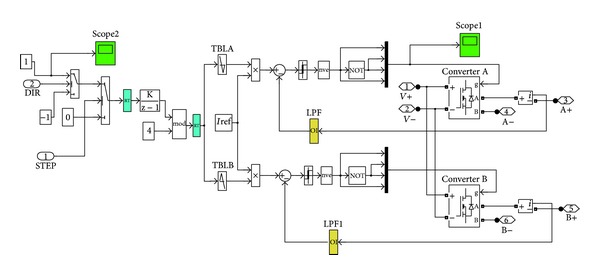
Schematic diagram of the stepper motor driver.

The infrastructure of the stepper motor driver incorporated in the overall model is graphically presented in Figure 4.

### 3.2. Wind Farm

The wind farm module, which is integrated in the overall simulation model developed, includes a Double fed induction generator (DFIG) wind turbine [12, 28] which incorporates pitch and power control. This specific DFIG unit is a 9 MW wind farm consisting of six 1.5 MW wind turbines connected to a 25 kV distribution system that exports power to a 120 kV grid through a 30 km, 25 kV feeder.

**Figure 5:**
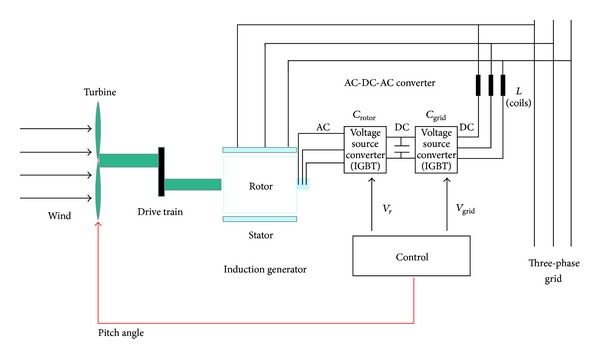
The DFIG wind turbine model adopted.

Figure 5 provides an illustrative overview of the DFIG model in which both pitch and power control are incorporated in the block named control.

The main principle of a DFIG is that the windings of the rotor are connected to the grid by the use of slip rings and back-to-back voltage source converter which regulates not only the rotor but also the volumes of the grid currents. As a result, rotor frequency may be independent from the grid frequency. The DFIG technology allows extracting maximum energy from the wind for low wind speeds by optimizing the turbine speed, while minimizing mechanical stresses on the turbine during gusts of wind.

During the simulation tests performed, the wind velocity was supposed to take values in the range from 6 m/sec (cut-in) to 30 m/sec (cut-off). Similarly, the nominal power per wind turbine reaches 1.5 MW.

Additionally, the generally applied theoretic formula which expresses the output power *P* of a wind turbine [28] was properly reformed in order to incorporate the yaw error signal due to yaw misalignment:

(1) $$\begin{array}{c} P=0.5\cdot \rho \cdot \pi \cdot R^{2}\cdot v^{3}\cdot C_{p}\left(\lambda \cdot \beta \right)\cdot n\cdot \cos\theta , \end{array}$$

where *ρ* symbolizes the air density, *R* stands for the effective radius of the turbine, *v* represents the mean value of the wind velocity at the height of the rotor axis, *C*_*p*_ is the so-called power coefficient or else aerodynamic efficiency, *λ* represents the tip velocity ratio of the rotor blade tip velocity to wind velocity, *β* symbolizes the blade pitch angle, *n* is the electromechanical efficiency, and *θ* expresses the yaw misalignment angle. Consequently, the wind turbine inner blocks were appropriately designed in order to conform to what (1) dictates.

### 3.3. Fuzzy Controller

The design of the fuzzy controller developed was based on a fuzzy inference system (FIS) and processes according to the analytical models described in [13, 23, 24].

More specifically, the FIS adopted is based on the model proposed by Takagi-Sugeno [29, 30]. The specific dynamic model utilizes a set of fuzzy rules in order to describe global nonlinear systems in terms of sets of local linear models which are smoothly connected by fuzzy membership functions.

The use of this fuzzy modeling method offers two main advantages. The first of them is that it provides a simpler yet effective alternative approach to powerful conventional control theory for the analysis and control of complex nonlinear systems. The second advantage is that Takagi-Sugeno models utilize a radically smaller number of control rules than other fuzzy models [31]. A synoptic list of the main FIS parameters utilized is given in Table 1.

The fuzzy controller developed incorporates three inputs, which, are namely, error, error change, and wind velocity. The output of the controller is the input signal to the stepper motor's driver. The driver converts this constant signal into a series of appropriate pulses, in the form illustrated in Figure 4, which consecutively drive the stepper motor.

The yaw error input signal is derived as the result of the subtraction of the wind direction angle minus the yaw motor angle. It is represented through the use of 7 membership functions. The range of angle values of each one of the membership functions corresponds to an acceptable yaw error angle for each of the three ranges of wind velocity which are described later on. The total range of angle values represented in this way is from −180° to 180°.

Additionally, the change of error signal provides information on both the relative deviation of the yaw error around the reference point and the velocity of this deviation. In a similar way, with the yaw error, the change of error signal is also represented through the use of 7 membership functions.

Furthermore, the current value of wind velocity is incorporated in the overall control model. This parameter is represented through the use of 3 membership functions which correspond to wind velocities in the ranges of 6–14 m/sec, 15–24 m/sec, and over 25 m/sec, respectively. The utilization of these input membership functions enables the full coverage of the total operational range of the wind turbine from the cut-in (6 m/sec) velocity to the cut-off (30 m/sec) velocity.

## 4. Simulation Setup and Results

### 4.1. Investigation of the Correlation between Wind Velocity and Acceptable Yaw Error

The first set of simulations performed took place without the use of the fuzzy controller, in order to explore the behavior of the wind turbine for different yaw angle errors.

The simulation scenarios examined refer to three different values of wind velocity, that is, 10 m/sec, 15 m/sec, and 25 m/sec (where the wind turbine is considered to operate properly).

For each one of these values of wind velocity, the power output of the wind turbine was measured while the yaw error was consecutively set to be equal to 0°, 3°, 5°, 10°, 15°, 20°, 30°, 45°, 50°, 60°, and 89°.

The results of the simulation tests which demonstrate the influence of the change of yaw error angle to the deviation wind turbine power output are summarized in Table 2.

The examination of the simulation data presented in Table 2 makes evident that the gradual increase of yaw error angle results in reduction of the power produced by the wind turbine. It is also shown that this reduction is nonlinear; that is, the rate of the decrease of power output is not the same with the magnitude of increase of error angle.

Furthermore, it becomes apparent that for every wind velocity there is a threshold beyond which the increase of yaw angle error causes a practically perceptible reduction of power output and thus the additional operation of a controller unit is necessitated in order to confront this effect. The underlined data in Table 2 indicate these specific cases.

**Figure 6:**
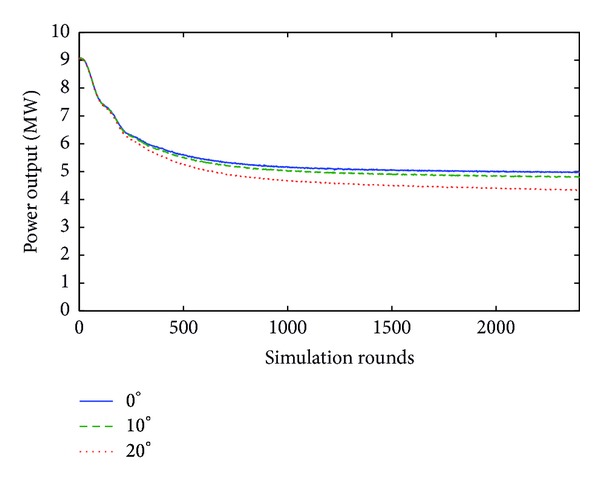
Power output for 0°, 10°, and 20° yaw error angle when wind velocity is equal to 10 m/s.

More specifically, referring to the case where *v* = 10 m/sec, although the rise of yaw error angle from 0° to 10° causes a relatively trivial reduction of power (by less than 4%), the further increase of yaw error angle by 10°, that is, from 10° to 20°, reduces power output down to 17.6% of the nominal value for this specific wind velocity. This can be graphically observed through Figure 6.

This behavior can be explained by taking into account the electrical and pitch angle control the wind farm module possesses; for example, when the power is high due to high wind speed and proper wind direction, the rotor speed is controlled by the pitch angle of the blades in order to smooth out power fluctuations.

**Figure 7:**
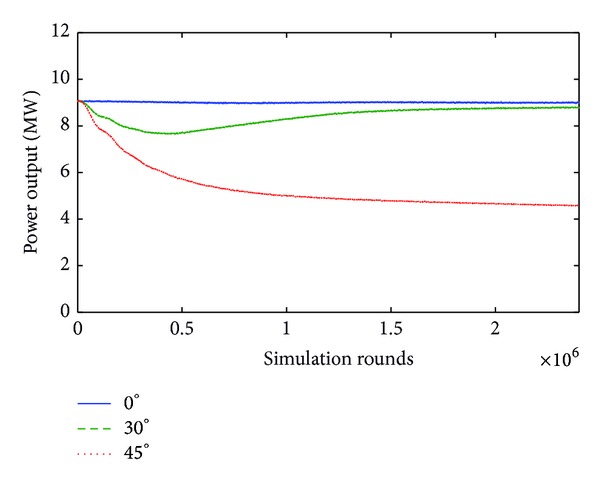
Power output for 0°, 30°, and 45° yaw error angle when wind velocity is equal to 15 m/s.

Similarly, when *v* = 15 m/sec, although the rise of yaw error angle from 0° to 30° reduces power by only 2.2%, the further increase of yaw error angle by 15°, that is, from 30° to 45°, decreases power output down to 48.9% of the nominal value for this specific wind velocity. This is depicted in Figure 7.

**Figure 8:**
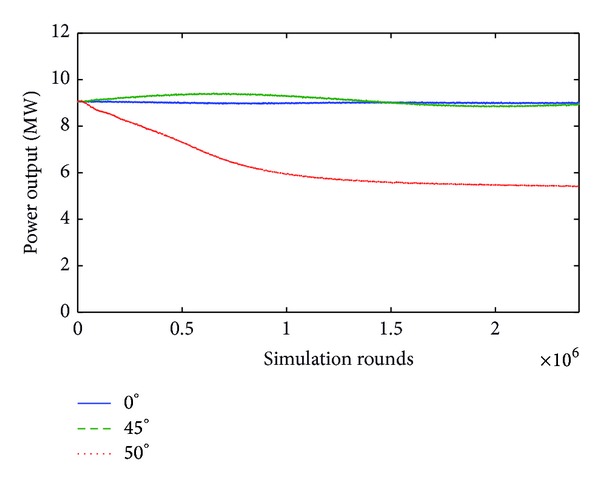
Power output for 0°, 45°, and 50° yaw error angle when wind velocity is equal to 25 m/s.

In the case where *v* = 25 m/sec, this effect becomes even more evident because although the rise of yaw error angle from 0° to 45° decreases power by just 1.1%, the further increase of yaw error angle by 5°, that is, from 45° to 50°, results in a power output drop down to 40% of the nominal value for this particular wind velocity. This is noticeably illustrated in Figure 8.

Therefore, based on these observations, it becomes profound that a yaw control regulator is necessitated, able to intervene adaptively to the existing values of wind velocity and yaw error angle.

### 4.2. Fuzzy Control Evaluation

The performance of the fuzzy controller developed was initially tested through simulation tests over three different values of wind velocity (i.e., 10, 15, and 25 m/sec) in order to check if the three levels of movement sensitivity are applied.

**Figure 9:**
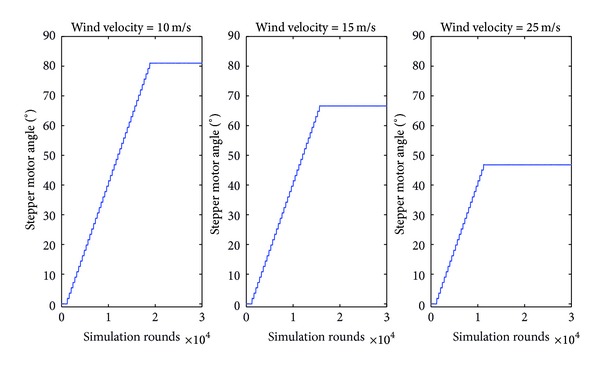
Yaw motor response on 90° input angle and 10, 15, and 25 m/sec wind velocities, respectively.

Figure 9 illustrates the different response of the yaw motor angle for each one of these wind velocities.

### 4.3. Full Model Simulation

Finally, the effectiveness of the proposed fuzzy yaw control system was investigated. This was achieved by setting up and conducting simulation tests in which the performance of the overall wind farm model was examined and validated under the same combinations of values of wind velocity and yaw error angle as the aforementioned.

The corresponding simulation results are summarized in Table 3. The observation of these simulation results confirms that the incorporation of the proposed control system enables the wind turbine to preserve high power output despite the increase of yaw error angle.

More precisely, these results indicate that the suggested fuzzy controller has successfully embedded three levels of yaw movement sensitivity.

**Figure 10:**
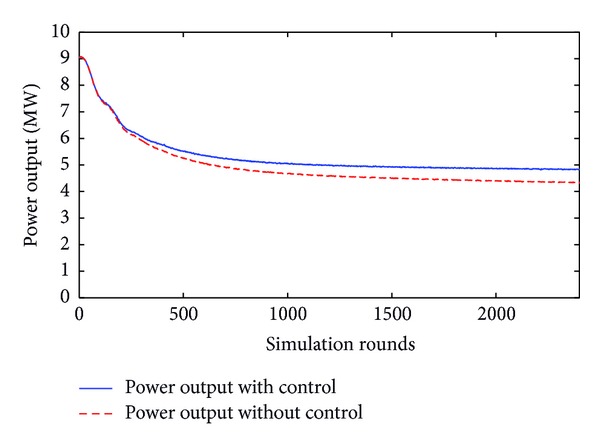
Power with and without yaw control for 20° error angle and 10 m/sec wind velocity.

The first level of sensitivity corresponds to wind velocities below 15 m/sec and performs yaw rotations only for errors greater than 15° angle. This is graphically presented in Figure 10.

**Figure 11:**
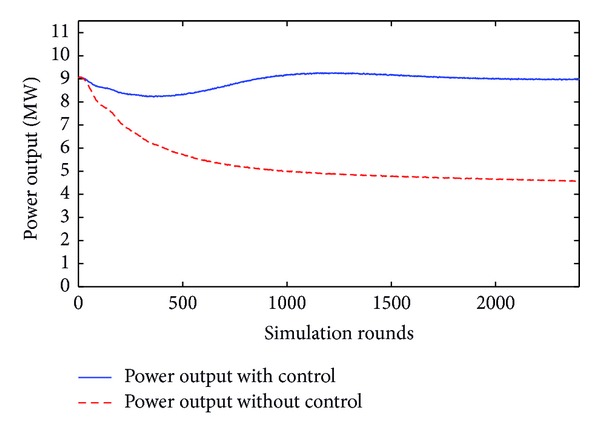
Power with and without yaw control for 45° error angle and 15 m/sec wind velocity.

Similarly, the second level of sensitivity refers to wind velocity ranging from 15 to 24 m/sec and yaw error being greater than 30°. This is illustrated in Figure 11.

**Figure 12:**
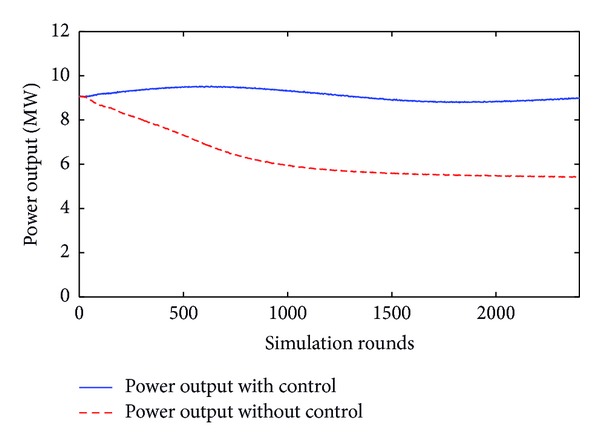
Power with and without yaw control for 50° error angle and 25 m/sec wind velocity.

In the same way, the third level of sensitivity corresponds to the case where wind velocity is higher than 25 m/sec and yaw error is greater than 45°. This is depicted in Figure 12.

It must be noted that the above plots begin with the nominal value of power output; that is, they refer to the steady state, because the imprint of simulation time before steady state is omitted.

This is due to the fact that this time is excessively extensive due to the long time constants of the different electromechanical parts and the slow regulators of the model. Fluctuations of power output that exceed the nominal value during the simulation are regulated by the pitch and power control systems of the wind turbine model.

It should also be noted that in order to change the acceptable yaw angle range or the wind velocity range for the levels of sensitivity, it is adequate to relocate properly the error input membership functions. The fuzzy rules are already tuned and do not need to be altered. Thus, the controller is easily adaptable to other models that operate in different environments.

Higher movement sensitivity results in both higher power output and faster wear of the system. Consequently, it is a matter of balance between the movement sensitivity and power output that each application should decide on.

## 5. Conclusions

In this research article, the wind turbine yaw control issue was investigated. An enhanced fuzzy controller has been designed to be able to regulate the yaw movement of wind turbines.

This novel controller has the ability to take into consideration both the direction and the velocity of wind and to appropriately adjust with a variable sensibility to this momentum.

This is the reason why the proposed wind turbine yaw control system achieves remarkable adaptation to the existing conditions.

Simulation tests performed demonstrate the capability of the proposed system to retain the wind turbine power output close to its nominal value and at the same time prohibit any unnecessary movement of the yaw system.

## Figures and Tables

**Table 1:** The fuzzy inference system parameters utilized.

Parameter	Type
FIS	Takagi-Sugeno
AND method	Min
OR method	Max
Implication method	Min
Aggregation method	Max
Defuzzification method	Wtaver

**Table 2:** Arithmetic presentation of power for various error angles and wind speeds without yaw control.

Error angle	Power output at steady state (MW)		
	*v* = 10 m/s	*v* = 15 m/s	*v* = 25 m/s
0°	5.1	9	9
3°	5.1	9	9
5°	5.1	9	9
10°	4.9	9	9
15°	4.8	8.9	9
20°	4.2	8.9	8.9
30°	3.7	8.8	8.9
45°	2.6	4.6	8.9
50°	2.3	3.6	5.4
60°	1.8	2.3	2.3
89°	0	0.1	0.2

**Table 3:** Arithmetic presentation of power for various error angles and wind speeds with yaw control.

Error angle	Power output at steady state (MW)		
	*v* = 10 m/s	*v* = 15 m/s	*v* = 25 m/s
0°	5.1	9	9
3°	5.1	9	9
5°	5.1	9	9
10°	4.9	9	9
15°	4.8	8.9	9
20°	4.8	8.9	8.9
30°	4.8	8.9	8.9
45°	4.8	8.9	8.9
50°	4.8	8.9	8.9
60°	4.8	8.9	8.9
89°	4.8	8.9	8.9
